# Neglected Inguinal Hernia Progressing to Strangulation: Surgical Implications and the Importance of Early Repair

**DOI:** 10.7759/cureus.90375

**Published:** 2025-08-18

**Authors:** Aldhi Tri Budhi, Febi Iswandi Suarno Tabi, Taufiq Qurrohman

**Affiliations:** 1 General Surgery, Dr. M. M. Dunda Limboto General Hospital, Gorontalo, IDN; 2 Department of Surgery, Faculty of Medicine, Universitas Negeri Gorontalo, Gorontalo, IDN

**Keywords:** bowel obstruction, hernia repair, inguinal hernia, lichtenstein repair, patient delay, strangulated hernia, surgical emergency

## Abstract

A strangulated inguinal hernia is a common surgical emergency with high morbidity and mortality. Patient delay in seeking treatment is a critical factor that increases surgical complexity and worsens prognosis. A 59-year-old male presented with a two-hour history of acute, excruciating pain in his left scrotum and signs of bowel obstruction. He had a two-year history of a neglected, reducible inguinoscrotal hernia. Examination revealed a strangulated hernia with systemic signs of distress, including severe hypertension (170/120 mmHg) and tachycardia (119 bpm). Emergency surgery confirmed a strangulated left inguinal hernia containing congested ileal loops and dense adhesions resulting from the chronic nature of the hernia. The bowel was successfully preserved after meticulous adhesiolysis, and a Lichtenstein mesh repair was performed. The patient had an uneventful postoperative course. Patient delay is the primary determinant of morbidity in strangulated hernias. The chronic presence of the hernia led to the formation of dense adhesions, significantly increasing the surgical complexity and operative risk compared to an elective repair. The management highlights the critical intraoperative assessment of bowel viability and the nuanced decision to use prosthetic mesh in a potentially contaminated field, aligning with current guidelines when bowel resection is not required. The case underscores that neglecting a hernia transforms a low-risk condition into a life-threatening emergency. Neglecting an inguinal hernia leads to preventable, life-threatening complications. This case emphasizes the critical importance of patient education to promote timely elective surgery to prevent the significant morbidity and mortality associated with strangulated hernias.

## Introduction

Inguinal hernias are among the most common conditions encountered in general surgery and are typically managed electively with low morbidity and recurrence rates. However, delayed presentation or neglect can result in strangulation, which significantly alters the clinical trajectory and transforms a manageable condition into a life-threatening emergency. Strangulated hernias comprise a substantial portion of abdominal surgical emergencies and are associated with high morbidity and mortality, especially when bowel resection is required due to ischemic complications [1,2]. Studies have shown that the risk of adverse outcomes in emergency hernia repair is markedly higher than in elective settings, with mortality rates increasing up to tenfold in elderly and comorbid patients [2-4].

Strangulation develops through progressive vascular compromise of herniated bowel loops, initially affecting venous outflow and eventually arterial inflow, resulting in ischemia and potential necrosis. Clinically, this presents with acute pain, irreducible swelling, and signs of systemic inflammation such as tachycardia and hemodynamic instability. The systemic impact of bowel ischemia is largely driven by endotoxin release and cytokine-mediated inflammatory cascades, which can precipitate sepsis or multi-organ failure if not managed promptly [3]. Rapid diagnosis and surgical intervention are therefore critical to minimizing systemic complications and preventing irreversible bowel damage.

Neglected or chronic hernias are often complicated by dense fibrous adhesions between the bowel and the hernia sac, as a result of repeated mechanical irritation and local inflammation over time. These adhesions increase intraoperative complexity, obscure normal anatomical planes, and elevate the risk of bowel injury during dissection [2,4]. Ceresoli et al. reported that delayed presentation was significantly associated with prolonged operative time, increased risk of bowel resection, and longer hospital stay in elderly patients undergoing emergency hernia surgery [4]. This supports the importance of early surgical referral for inguinal hernias, even in asymptomatic cases.

A key area of debate in the emergency management of strangulated hernias is the safety of mesh implantation in the presence of potential contamination. Historically, the use of synthetic mesh was avoided due to concerns about surgical site infections and mesh-related complications. However, emerging evidence challenges this paradigm. Marcolin et al. demonstrated that mesh repair in incarcerated and strangulated hernias, when performed without gross contamination, reduced recurrence without increasing infection risk [5]. Similarly, Duan et al. showed that polypropylene mesh could be safely used in cases with Grade I bowel necrosis, provided that bowel viability was restored and no perforation occurred [6]. Nonetheless, the Cochrane review by Sæter et al. emphasized the heterogeneity and limitations of available studies, highlighting the need for cautious, case-by-case decision-making [7].

This case report presents a 59-year-old male with a long-standing inguinoscrotal hernia who developed acute strangulation following two years of neglect. The case highlights several critical issues: the deleterious effects of patient delay, the technical challenges posed by chronic adhesions, and the nuanced decision-making process surrounding emergency mesh repair. By reviewing current evidence and detailing the intraoperative approach, this report aims to underscore the importance of early surgical intervention and provide insight into best practices for managing complex strangulated hernias [1-9].

## Case presentation

A 59-year-old male market vendor presented to the Emergency Department with a two-hour history of sudden, excruciating left scrotal pain, rated 10/10. The pain was accompanied by a newly firm, tender scrotal swelling. The patient reported that this mass had been present for two years, initially painless, reducible, and more prominent during physical exertion, for which he had not sought medical attention. He also reported an inability to pass stool since that morning, associated with severe diffuse abdominal pain. The scrotal mass was irreducible and demonstrated no cough impulse. 

**Figure 1:**
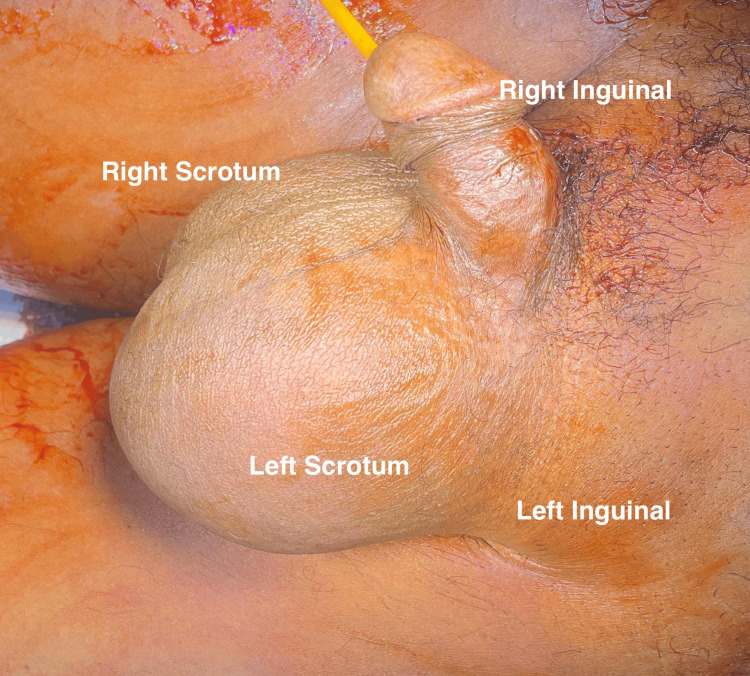
Left inguinoscrotal hernia with strangulation Left groin and scrotum revealed a large, tense, and exquisitely tender mass extending from the inguinal canal into the scrotum.

Upon examination, the patient was conscious but appeared to be in moderate distress. His vital signs were notable for hypertension (170/120 mmHg), tachycardia (119 beats per minute), tachypnea (20 breaths/min), and mild hypoxia (SpO₂ 91% on room air). Abdominal examination revealed distension and diffuse tenderness. The severe abdominal pain was attributed to visceral irritation from the incarcerated hernia and associated ileus, rather than the short history of constipation alone. Examination of the left groin/scrotum confirmed a large, tense, exquisitely tender mass extending from the inguinal canal into the scrotum (consistent with an incarcerated and likely strangulated hernia as seen in Figure 1). Initial labs revealed an elevated hemoglobin (17.0 g/dL), suggestive of hemoconcentration secondary to dehydration or acute stress. Given the unequivocal clinical signs of a strangulated hernia mandating immediate surgical intervention, no preoperative abdominal imaging (X-ray or CT) was obtained to avoid delaying time-critical management. All other initial laboratory results were within normal limits, as shown in Table 1.

**Table 1:** Laboratory examination result prior to emergency surgery The results show that the patient has a slightly increased hemoglobin of 1 g/dL above the upper treshold for male hemoglobin, while the other laboratory indicators within normal limits.

Test name	Result	Reference range	Unit
CT (clotting time)	11'	9-15	Minutes
BT (bleeding time)	2'	1-3	Minutes
Random blood glucose	112	<200	mg/dL
HBs Ag	Non-reactive	-	-
Hemoglobin	17.0	12-16	g/dL
Hematocrit (male)	48.5	42-54	%
Erythrocytes	5.50	3.5-5.5	Million/mm³
Leukocytes	8.5	5-10	Thousand/mm³
Platelets	381	150-450	Thousand/mm³

**Figure 2:**
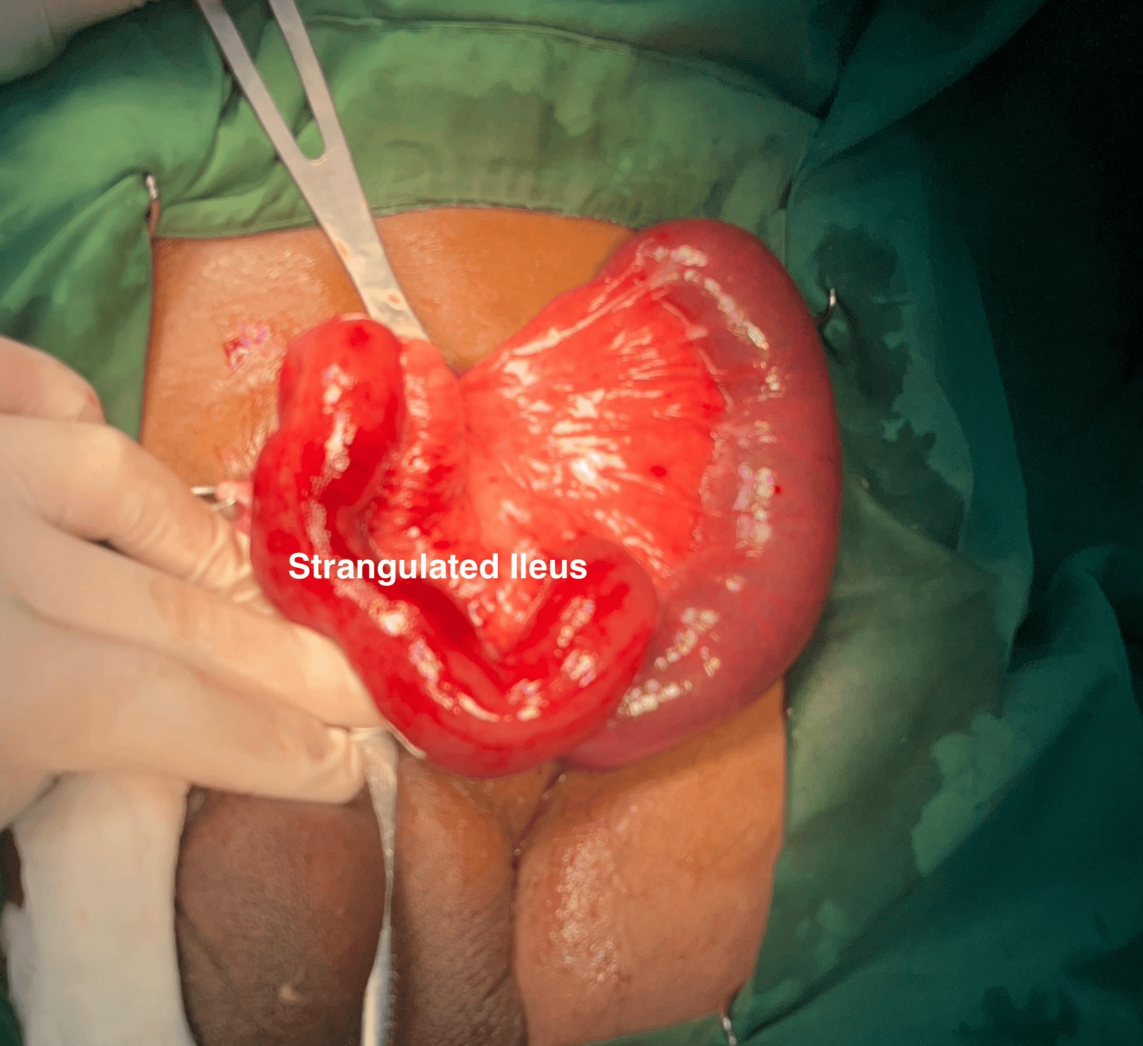
Intraoperative findings of the strangulated ileum during surgery The affected bowel appears congested, dark red to purple, and edematous, indicating venous congestion and early signs of ischemic injury. The surrounding tissue is being retracted for better visualization during the surgery.

The patient was taken for emergency surgery with a preoperative diagnosis of a strangulated left inguinal hernia. Upon surgical exploration via an inguinal incision, the hernia sac was found to be filled with edematous and deeply congested loops of the ileum, as shown in Figure 2. The chronic nature of the hernia was immediately apparent from the presence of dense, fibrous adhesions between the intestinal loops and the inner wall of the hernia sac. These adhesions were a direct result of the long-standing, neglected hernia. The surgeon performed a meticulous and time-consuming adhesiolysis to carefully free the entrapped bowel without causing injury.

The operative photos document the critical stages of the procedure. One image shows the exteriorized loop of the ileum immediately after release; it appears swollen and dark purple (violaceous), which are classic signs of severe venous congestion and ischemia from strangulation, as shown in Figure 2. However, the serosal surface still retained its sheen, an important indicator of potential viability. After freeing the bowel, it was wrapped in warm, saline-soaked gauze. Over several minutes, the color improved to a healthy pink, and peristalsis was observed, confirming its viability. The viable intestine was then reduced back into the abdominal cavity.

**Figure 3:**
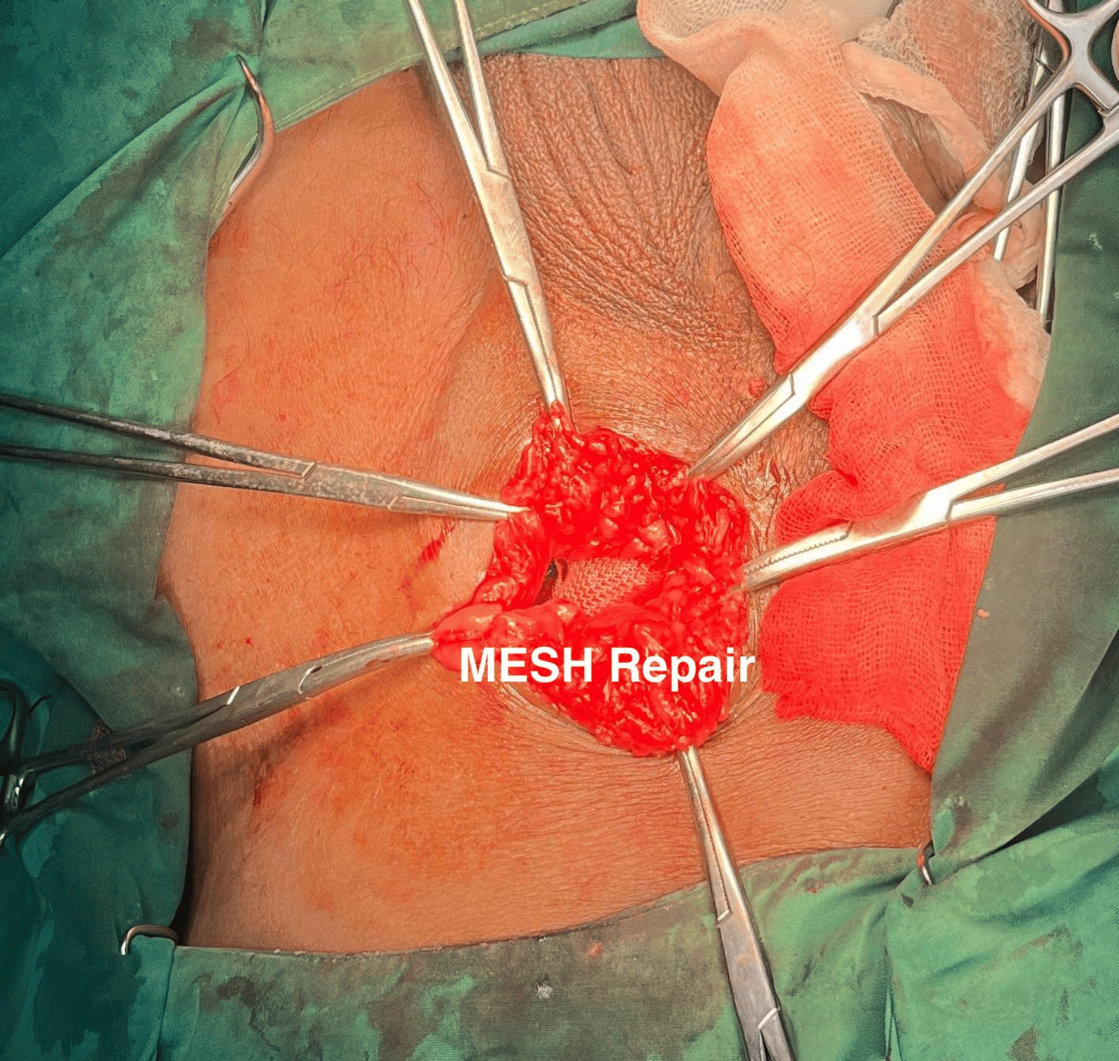
Intraoperative closure of the hernia defect In the center, a polypropylene mesh is visible and has been placed over the hernia opening. This mesh reinforces the weakened area to prevent recurrence. The surrounding tissues have been prepared for suturing the mesh into place, a standard part of tension-free hernia repair techniques.

A herniotomy was performed, and the inguinal floor was reconstructed using the tension-free Lichtenstein technique with a polypropylene mesh, as shown in Figure 3. The final postoperative diagnosis was a strangulated left inguinal hernia with intestinal adhesions, successfully managed with adhesiolysis and Lichtenstein herniorrhaphy. The patient was instructed to remain fasting until the return of bowel function, and he had an uneventful postoperative recovery. The patient resumed oral intake on postoperative day 2 and was discharged on day 4. At the three-month follow-up, no recurrence, chronic pain, or mesh-related complications were noted

## Discussion

This case of a neglected inguinoscrotal hernia culminating in acute strangulation underscores several critical clinical considerations. While our patient presented promptly (within two hours) once acute, excruciating pain developed - a crucial factor likely contributing to the viability of the incarcerated bowel and avoiding the need for resection - the preceding two-year delay significantly increased operative complexity. Chronic, untreated hernias often develop dense fibrous adhesions between the herniated contents and the sac wall [1], as observed intraoperatively in this patient. These adhesions obscure anatomical planes, increase tissue friability, and substantially elevate the risk of iatrogenic injury during dissection compared to elective repairs performed on non-inflamed tissues. 

This delay was driven by significant socioeconomic barriers inherent to his context as a market vendor in a resource-limited setting: financial constraints limiting access to care, fear of lost income during recovery, and restricted availability of timely, affordable surgical services. This exemplifies the well-documented association between prolonged hernia neglect and increased morbidity, including higher bowel resection rates and worse prognosis [2-4]. The initial laboratory finding of elevated hemoglobin (17.0 g/dL) likely reflected hemoconcentration secondary to dehydration (potentially from reduced oral intake due to pain or vomiting) or acute stress polycythemia, rather than a primary hematological disorder [5]. It is also important to note the variant known as Richter's hernia, where only part of the bowel circumference is entrapped. Although not present in this case, Richter's hernia can present with strangulation and necrosis without obvious intestinal obstruction, potentially leading to diagnostic delay [6].

The pathophysiology of strangulation involves initial venous outflow obstruction from the entrapped bowel segment, progressing to arterial compromise, ischemia, and potential necrosis. Systemic release of inflammatory mediators from ischemic or necrotic bowel can trigger a profound inflammatory response, potentially evolving into SIRS. Our patient's presenting vital signs (hypertension, tachycardia, tachypnea, and mild hypoxia) were multifactorial: primarily driven by ischemia-induced inflammatory cascades [3], but significantly compounded by excruciating pain (10/10) and acute anxiety. Prompt surgical intervention remains paramount to halt this progression and prevent multi-organ failure. Regarding imaging, while CT scanning or abdominal X-ray is often pursued for acute abdominal presentations and can be valuable (e.g., confirming obstruction, assessing bowel viability remotely, or evaluating for Richter's hernia), the unequivocal clinical signs of a strangulated hernia in this patient mandated immediate surgical exploration [6]. Obtaining imaging would have delayed definitive, life-saving treatment without altering the urgent management plan.

Our patient's lack of significant medical comorbidities was a favorable factor, likely mitigating his risk profile despite requiring emergency surgery. Emergency hernia repair, particularly in elderly patients or those with comorbidities, carries a substantially higher risk of complications and mortality compared to elective repair, often due to ischemia, resection needs, and the physiological stress of acute illness compounded by surgical delay [4]. Ceresoli et al. reported a two-to-10-fold increase in mortality in elderly emergency patients [4]. In this case, the patient's relative health likely contributed to his ability to withstand the physiological insult and the complex dissection required due to the chronic adhesions encountered.

The intraoperative decision regarding mesh use in potentially contaminated fields is complex and context-dependent. In this specific case, the bowel, although initially appearing congested, regained normal color and peristalsis after warm saline application, confirming viability with no evidence of perforation or gross contamination (CDC wound class I: clean-contaminated). Based on this assessment, the surgical team opted for a Lichtenstein repair using polypropylene mesh. While mesh repair is generally considered acceptable in carefully selected Grade I (clean-contaminated) cases without bowel resection or perforation [7,8], its use in higher-grade contamination (CDC class II/III/IV) requires extreme caution due to significantly elevated risks of surgical site infection, mesh infection, chronic sinus formation, and the need for explantation. Long-term infection data for mesh in even Grade I emergency settings remain limited. Systematic reviews caution that the evidence base is limited by study quality and sample size [8]. Therefore, we present this choice as the specific intraoperative judgment made for this patient under favorable local conditions (viable bowel, no perforation, Grade I), not as a general recommendation. Close long-term follow-up is essential for patients undergoing emergency mesh repair to monitor for recurrence, chronic pain (CPIP), and mesh-related complications such as infection or migration; in this case, no recurrence or complications were noted at the three-month follow-up [9].

Addressing the global health challenge of delayed presentation requires moving beyond description to implementing solutions. Strategies could include 1) community-based hernia screening and education programs to identify reducible hernias early, 2) developing subsidized pathways or financing mechanisms for elective hernia repair targeting low-income populations like market vendors, 3) task-sharing models training general medical officers in basic hernia repair to increase service availability in rural/peripheral areas, and 4) streamlining referral systems to reduce wait times for elective surgery. In resource-limited settings, factors such as direct costs (consultation, surgery), indirect costs (transportation, lost wages during recovery), and limited healthcare infrastructure significantly contribute to delayed presentation [8-11].

Delaying elective hernia repair significantly increases the risks of strangulation, complex emergency surgery, and worse long-term outcomes, including longer hospital stays, increased CPIP, nerve injury risk, psychological distress, and delayed return to function [11]. This patient's emergency presentation and the subsequent challenging repair starkly illustrate these consequences. Postoperative recovery in this case was uncomplicated: the patient resumed oral intake on postoperative day 2 and was discharged on day 4. At the three-month follow-up, no recurrence, chronic pain, or mesh-related complications were noted.

## Conclusions

The successful management of this strangulated inguinoscrotal hernia resulted from prompt presentation after acute symptom onset (facilitating bowel salvage), timely surgery, and the patient's lack of comorbidities. The case highlights critical intraoperative assessments (bowel viability) and nuanced mesh decision-making in a controlled Grade I scenario. The preventable two-year delay was driven by socioeconomic barriers (financial constraints, fear of income loss, and healthcare access limitations) rather than neglect, reflecting systemic challenges in resource-limited settings. This underscores the urgent need for multifaceted interventions, namely, 1) patient education on hernia risks, 2) improved access to affordable elective surgery, and 3) systemic support for vulnerable workers, to prevent such high-acuity emergencies.
